# Unicompartmental knee arthroplasty after failed high tibial osteotomy in appropriately selected patients: a systematic review and meta-analysis

**DOI:** 10.1186/s42836-026-00434-2

**Published:** 2026-10-05

**Authors:** Fardis Vosoughi, Mobina Taghva Nakhjiri, Amir Arshia Saghafi, Faryar Koolinia, Golnaz Nikeghbali, Iman Menbari Oskouie

**Affiliations:** 1 Department of Orthopaedics and Trauma Surgery, Shariati Hospital, Tehran University of Medical Sciences, Tehran, 1411713135 Iran; 2 Imam Khomeini Hospital Complex, Tehran University of Medical Sciences, Tehran, 1419733141 Iran; 3 Student Research Committee, School of Medicine, Tehran University of Medical Sciences, Tehran, 1416634793 Iran; 4 Student Research Committee, School of Medicine, Shahid Beheshti University of Medical Sciences, Tehran, 1983963113 Iran; 5 Sports Orthopaedics and Arthroscopy Research (SOAR) Center, Tehran, Iran

**Keywords:** High tibial osteotomy, Failed HTO, Conversion, UKA, TKA, Implant sizing, Systematic review, Meta-analysis

## Abstract

**Aims:**

High tibial osteotomy (HTO) is an established treatment for medial knee osteoarthritis in younger, more active patients than typical arthroplasty candidates, but some may require conversion over time. Total knee arthroplasty (TKA) has traditionally been the preferred procedure after failed HTO. With advances in implant design and surgical techniques, the role of medial unicompartmental knee arthroplasty (UKA) in this setting warrants re-evaluation. This systematic review and meta-analysis aims to summarize and descriptively compare the outcomes of UKA and TKA following HTO.

**Methods:**

A systematic review and meta-analysis was conducted on MEDLINE, Embase, Scopus, and Web of Science until 14th August 2025 (date last searched). Randomized and observational studies (cohort, case-control, and case series) reporting outcomes of UKA or TKA following HTO were included, while case reports, technical notes, reviews, and studies without sufficient data were excluded.

**Results:**

Forty-one studies (6 UKA-only, 33 TKA-only, and 2 comparative studies contributing to both groups) met inclusion criteria. Due to the limited number of comparative studies, comparisons between procedures are indirect/descriptive and should not be interpreted as evidence of relative treatment performance. After HTO, UKA demonstrated pooled revision and reoperation rates of 10.2% (95% CI = 4.5–21.6%, I^2^ = 61.6%) (in 226 knees) and 14.4% (95% CI = 9.1–21.9%, I^2^ = 37.0%) (in 226 knees), respectively, with PJI rates of 0–3.3% (in 71 knees). Mean improvement in the Knee Society Score (KSS) ranged from 34.09 to 38.80 points. For TKA, the pooled revision, reoperation, and PJI rates were 5.0% (95% CI = 3.5–7.2%, I^2^ = 79.5%) (in 6208 knees), 11.1% (95% CI = 7.3–16.3%, I^2^ = 92.8%) (in 6236 knees), and 2.9% (95% CI = 2.0–4.0%, I^2^ = 33.5%) (in 2107 knees), with a KSS improvement range of 25.0 to 57.0 points. Direct comparative studies found no significant differences in survivorship or function. In an exploratory analysis of studies published after 2020, the revision rate for UKA and TKA was 6.3% and 3.9%, respectively. The most common cause of UKA revision was lateral progression of osteoarthritis. The meta-analysis showed a pooled infection rate of 2.9% (in 2107 knees) following TKA.

**Conclusions:**

This study suggests that UKA after HTO should not be considered an absolute contraindication and may have a role in a carefully selected subset of patients with isolated medial osteoarthritis, sufficient MCL, and acceptable residual alignment. However, these findings should be interpreted cautiously, as the current evidence base consists predominantly of retrospective observational studies. Accordingly, the present findings should be viewed as hypothesis-generating rather than as evidence establishing equivalence or comparative effectiveness between UKA and TKA after failed HTO. Prospective comparative studies are needed to better define its effectiveness relative to TKA, refine patient selection algorithms, and clarify the influence of residual deformity on outcomes.

**Level of evidence:**

Level III, systematic review and meta-analysis.

**Supplementary Information:**

The online version contains supplementary material available at https://doi.org/10.1186/s42836-026-00434-2.

## Introduction

High Tibial Osteotomy (HTO) is an option for the treatment of medial osteoarthritis (OA) due to varus alignment in younger or active patients (40–60 years) [1–3]. The short- and mid-term survival of HTO is generally satisfactory (91–98%) [4], with high patient satisfaction [5]. However, long-term durability is limited [6], with 11.1% conversion rates to arthroplasty after 10 years from HTO [7].

Primary UKA-related literature suggests a significantly reduced rate of thromboembolic events (0.28% vs. 0.44%), cardiovascular events (0.13% vs. 0.21%), periprosthetic joint infection (PJI) (0.82% vs. 0.84%), and mortality (0.06% vs. 0.12%) compared to TKA [8]. These advantages, along with bone preservation, more natural knee kinematics, and higher patient satisfaction, especially in younger patients [9], make UKA a favorable option for patients with severe medial OA.

Nevertheless, for those with a history of failed HTO, UKA has been historically deemed not suitable mainly due to concerns regarding the overcorrection of the knee, altered tibial anatomy and bone stock, changes in tibial slope, ligament balance disturbance, component positioning difficulties due to prior osteotomy, and retained hardware [10]. Therefore, when conversion becomes necessary, TKA has traditionally been the preferred solution for HTO failure [11]. With recent advancements in implant design and surgical techniques, many of the earlier limitations are being revisited, prompting a re-evaluation of UKA as a possible option for a highly selected group of patients following HTO [12]. Meanwhile, the cited lower complication rates from primary UKA-related literature may not directly apply to the post-HTO conversion setting.

The previous systematic review on this subject did not include recent data or comparison with TKA [13]. Therefore, this systematic review and meta-analysis was conducted to update and expand the available evidence by evaluating clinical outcomes after both UKA and TKA following failed HTO. Specifically, we aimed to systematically summarize revision and complication rates, functional outcomes, and radiological measures. The findings are intended to present the best available descriptive evidence to inform surgical decision-making in this patient population, rather than to establish definitive comparative effectiveness between procedures.

## Materials and methods

### Protocol and registration

This study was prospectively registered in the International Prospective Register of Systematic Reviews (PROSPERO; registration ID: CRD420251113089). The study was designed and reported under the Preferred Reporting Items for Systematic Reviews and Meta-Analyses (PRISMA) 2020 guidelines [14].

### Search strategy

A comprehensive literature search was performed following the PRISMA extension [15] for search strategies. Four electronic databases, MEDLINE/PubMed, Scopus, Web of Science, and Embase, were searched from their inception until 14th August 2025. Each database strategy was adapted individually and included free-text keywords and synonyms related to “knee arthroplasty” and “tibia osteotomy”. The final search strategy was reviewed by a specialist in knee surgery (initials blinded for review) using the PRESS (Peer Review of Electronic Search Strategies) [16] framework to ensure accuracy and completeness. Additionally, the reference lists from relevant studies were manually reviewed to identify additional publications. The search strings used for each database are provided in Supplementary Material 1.

### Eligibility criteria

Inclusion and exclusion criteria were based on the PICOS framework:

- Population: All patients who underwent knee arthroplasty (UKA or TKA) following HTO.
- Intervention: Medial UKA performed following a failed HTO. Both fixed-bearing and mobile-bearing implant designs were eligible.
- Comparator: Primary TKA performed following a failed HTO.
- Outcomes: Patient-reported outcome measures (PROMs), radiologic measures, reoperation, complications, and failure rates.
- Study design: randomized controlled trials, prospective or retrospective cohort studies, case-control studies, cross-sectional, and case series.

A failed HTO was defined as any prior HTO that subsequently required conversion to knee arthroplasty (UKA or TKA), regardless of the underlying indication. All forms of HTO were eligible, including medial opening-wedge and lateral closing-wedge techniques, irrespective of fixation method, retained hardware status, or whether hardware removal was performed before or during arthroplasty. Double-level osteotomies were excluded when separate reporting of outcome was not available. Revision arthroplasty procedures were not considered index interventions and were recorded only as outcome events when reported. We included studies irrespective of their publication year or language. We excluded technical notes, reviews, case reports, in vitro studies, conference abstracts, editorials, commentaries, animal or cadaveric studies, and studies without sufficient data for analysis.

### Study selection and data extraction

All search results were imported into EndNote version 9 (Clarivate Analytics, Philadelphia, PA, USA) [17], and duplicates were removed. The remaining records were uploaded into Rayyan (Qatar Computing Research Institute, Doha, Qatar), a web-based tool for screening [18]. Two reviewers (initials blinded for review) independently screened titles and abstracts, followed by full-text evaluation of potentially relevant studies. Disagreements at any stage were resolved by consensus with a third reviewer (initials blinded for review).

Data extraction was carried out using a standardized spreadsheet, piloted beforehand with the supervision of the senior author. Extraction was performed independently by two researchers (initials blinded for review). The following data items were considered to be collected: study characteristics (author, year, country, study design, and sample size), patient demographics (age, sex, body mass index, and affected knee), surgical method (UKA or TKA), follow-up duration, and complications (infection, revision, and reoperation). Clinical outcomes included knee range of motion (ROM), Knee Society Score (KSS), Hospital for Special Surgery (HSS) score, Oxford Knee Score (OKS), the University of California Los Angeles (UCLA), and Western Ontario and McMaster Universities Osteoarthritis Index (WOMAC). Radiographic outcomes comprised the hip–knee–ankle (HKA) angle and the Insall–Salvati ratio.

The outcomes of interest in the current review were revision/survivorship and complication rates, patient-reported outcome measures (PROMs), and functional and radiological outcomes of the patients. Revision was consistently defined as re-arthroplasty (e.g., conversion or exchange of prosthetic components), while reoperation was defined as any additional surgical procedure performed in the operating room under anesthesia (including revision surgeries).

### Risk of bias assessment

The methodological quality of included studies was assessed according to the study design. For non-randomized studies, the Risk of Bias In Non-randomized Studies of Interventions (ROBINS-I, version 2) [19] tool was used, which evaluates bias across seven domains: (1) confounding, (2) classification of interventions, (3) selection of participants, (4) deviations from intended interventions, (5) missing data, (6) measurement of outcomes, and (7) selection of the reported results. Each domain was judged as “low,” “moderate,” or “serious risk of bias.” The methodological quality of the included non-comparative studies was assessed using the Methodological Index for Non-Randomized Studies (MINORS), which was developed and validated for the appraisal of non-randomized surgical and interventional studies, including non-comparative case series [20]. The non-comparative version of the MINORS instrument consists of eight items, each scored as 0 (not reported), 1 (reported but inadequate), or 2 (reported and adequate), yielding a maximum score of 16. Two reviewers independently evaluated the risk of bias for each included study across the predefined domains. Discrepancies in judgments were resolved by the decision of a third reviewer. The Robvis tool (McGill University, Montreal, QC, Canada) [21] was used to visualize the results of the ROBINS-I version 2, while the individual item scores of MINORS are presented in Supplementary Table S1.

### Meta-analysis

All analyses were conducted in R software version 4.4.3 (R Foundation for Statistical Computing, Vienna, Austria) [22] using the meta package. In conducting the meta-analysis, we applied predefined criteria to ensure the interpretability of pooled estimates. Meta-analyses were performed only when sufficient data were available across studies. Outcomes reported in fewer than three studies were excluded from quantitative analysis due to limited data and high potential for bias. In such cases, findings were instead summarized qualitatively. All pooled outcome analyses were performed at the knee level. The outcome-specific denominators were derived by summing the number of knees from the individual studies reporting the corresponding outcome (e.g., revision, reoperation, or infection). The number of knees included in each study is presented in Tables 1 and 2, allowing the denominators used for each pooled analysis to be readily verified and ensuring complete traceability of the reported estimates.

**Table 1:** Baseline characteristics and outcomes of the UKA group

**Author**	**Year**	**Country**	**Study design**	**Indications**	**Prosthesis**	**No. Knees**	**No. Patients**	**Gender (male)**	**Age (mean ±SD)**	**BMI (mean ± SD)**	**Follow-up, months (mean** ±** SD)**	**Reoperations**	**Revisions**	**Infections**	**Complications**
J.L.Rees	2001	England	Prospective case-control	- isolated anteromedial primary OA - intact ACL - full-thickness lateral articular cartilage	NR	18	18	NR	68 ± 8.0	NR	64.8	5	5	NR	5
P.Vorlat	2006	Belgium	Retrospective comparative	- isolated medial OA - varus < 15° and manually correctable	NR	8	8	NR	NR	NR	66.4 ± 35.2	4	4	NR	4
T.J.Heyse	2012	Germany	Retrospective comparative	- able to localize pain on medial side - patellofemoral OA acceptable if silent and not bone-on-bone - absence of advanced lateral OA - range of motion > 100° - flexion deformity ≤ 15° after notch plasty and removal of the anterior bony block of the tibial spine - clinically stable knee in frontal and sagittal planes - less than 6° valgus and 15° varus	Fixed-bearing	18	18	NR	53.7 ± 5.8	NR	120.0 ± 55.2	2	2	NR	2
G. A.Valenzuela	2013	USA	Retrospective case–control	All patients with radiologic and symptomatic progression of isolated medial OA	Fixed-bearing	22	20	13	64.8 ± 8.0	27.4 ± 5.5	77.6 ± 39.0	3	1	NR	3
M.Schlumberger	2020	Germany	Retrospective case series	- medial OA (grade 3–4 KL) - intact ACL - no flexion contracture - no malalignment ≤ 15° - intact lateral compartment confirmed with MRI - intact ligament status confirmed with MRI	Mobile-bearing	30	30	25	59.5 ± 9.1	23.9 ± 4.0	51.6 ± 31.2	4	2	1	5
A.Parente	2021	Italy	Retrospective case–control	- BMI < 30 - isolated unicompartmental OA - varus < 10° - tibial slope < 10° - absence of patellofemoral pathology - absence of ligamentous incompetence	Fixed-bearing	26	26	13	66.0 ± 7.2	27.1 ± 6.5	93.6 ± 33.0	2	2	NR	0
J.C.Waï-Nam	2023	France	Retrospective case series	Medial knee OA with: - intact lateral compartment, - absence of patellofemoral pain - coronal plane deformity < 15°	Mobile-bearing	41	38	NR	61.0 ± 9.0	NR	79.0 ± 43.0	6	4	1	6
C. Schweizer	2025	Germany	Retrospective cohort	end-stage medial OA with: - preserved joint line - no excessive valgus (HKA < 5° and MPTA < 95°) - Oxford indications - fixed flexion contracture of less than 15° - no lateral patellar bone loss, grooving, or maltracking	Mobile-bearing or Fixed-bearing	63	60	37	61.0 ± 8.0	28 ± 5.0	60.0 ± 12.0	5	2	NR	NR

*Abbreviations: SD* standard deviation, *OA* osteoarthritis, *ACL* anterior cruciate ligament, *BMI* body mass index, *MRI* magnetic resonance imaging, *HKA* hip-knee-ankle angle, *MPTA* medial proximal tibial angle, *NR* not reported

**Table 2:** Baseline characteristics and outcomes of the TKA group

**Author**	**Year**	**Country**	**Study_design**	**No. Knees**	**No. Patients**	**Age (mean** ±** SD)**	**BMI (mean** ±** SD)**	**Follow-up, months (mean** ±** SD)**	**Reoperation**	**Revision**	**Infection**	**Readmission**	**Complication**
J.W.Staeheli	1987	USA	Case–control	35	35	67.3 ± 9.7	NR	44 ± 11.5	9	1	1	NR	NR
A.Amendola	1989	Canada	Case–control	42	39	64 ± 7.5	NR	NR	4	2	1	NR	5
T.Gill	1995	USA	Case–control	30	27	65 ± 5.5	NR	NR	6	1	NR	NR	5
Sören Toksvig-Larsen	1998	Sweden	Retrospective cohort	40	40	69 ± 6.0	NR	NR	4	4	NR	NR	NR
F.S.Haddad	2000	England	Case–control	50	42	NR	NR	NR	15	2	1	2	NR
J.B.Meding	2000	USA	Case–control	95	82	66.2 ± 8.7	NR	103.2 ± 45	14	1	6	NR	11
T.Noda	2000	Japan	Retrospective cohort	28	23	69.4	NR	25 ± 27.5	NR	NR	NR	NR	7
Bob Karabatsos	2002	Canada	Retrospective cohort	20	20	64 ± 8.2	NR	62.4	1	0	1	NR	3
P.Haslam	2007	England	Case–control study	51	40	78	NR	151.2 ± 30.0	31	8	3	NR	34
K.J.Kazakos ·	2008	Greece	Retrospective cohort	38	32	67.2 ± 7.0	NR	Not reported	NR	NR	1	NR	1
Y.Akasaki	2009	Japan	Case series	20	20	72 ± 7.0	NR	61.2 ± 14.7	0	0	NR	NR	NR
D.K.Bae	2010	Korea	Retrospective cohort	16	13	64.9 ± 6.2	27.22	56.4 ± 30.9	NR	NR	NR	NR	NR
H.Orban	2011	Romania	Case series	16	16	64.3	25.8	51.6	NR	NR	NR	NR	NR
S.Treuter	2012	Germany	Case series	48	48	NR	NR	159.6 ± 16.5	1	NR	NR	NR	3
Luis A. Farfalli	2012	Argentina	Retrospective cohort	34	31	67 ± 9.0	NR	96 ± 51.7	16	8	3	NR	12
H.Gupta	2013	India	Retrospective cohort	58	55	61.9 ± 7.5	29.6 ± 3.2	134.4 ± 45.0	11	4	4	NR	10
P.Hernigou	2013	France	Case–control	50	50	65 ± 6.7	NR	180 ± 30.0	2	2	NR	NR	2
G.A.Valenzuela	2013	USA	Case–control	18	15	64.2 ± 10.2	33 ± 4.5	NR	4	2	NR	NR	4
T.Niinimaki	2014	Finland	Case–control	1036	1036	64.3 ± 14.2	NR	80.4 ± 66.0	93	93	14	NR	NR
A.Dahl	2016	Sweden	Case–control	119	119	63 ± 7.7	29.1 ± 3.5	NR	5	5	NR	NR	NR
D.K.Bae	2017	South Korea	Case–control	32	29	68.3 ± 6.2	26.6 ± 2.4	74.4 ± 48.0	0	0	NR	NR	0
J.B.T.Lim	2017	Singapore	Case–control	217	217	64.5 ± 7.3	28.9 ± 5.0	24	15	3	8	NR	15
M.Ehlinger	2017	France	Retrospective cohort	135	135	63.2	NR	88.9 ± 32.5	16	11	2	NR	11
X.P.Carnero	2018	Spain	Case–control	41	41	70 ± 8.0	31.2	145.2	NR	NR	0	NR	0
B.Chalmers	2019	USA	Case–control	231	207	64 ± 12.2	31 ± 8.0	96 ± 45	52	20	5	NR	14
Anders El-Galaly	2019	Denmark	Retrospective cohort	1155	1155	63 ± 14.5	NR	111.6 ± 74.6	101	101	NR	NR	18
C.Batailler	2020	France	Case–control	41	41	65.4 ± 8.4	31.4 ± 4.1	93.6 ± 28.8	1	1	2	NR	9
A.Parente	2021	Italy	Case–control	33	33	74 ± 11.2	25 ± 2.1	134.4 ± 36	4	2	NR	NR	0
C.A.Primeau	2021	England	Retrospective cohort	103	103	50.2 ± 6.7	31 ± 5.6	NR	NR	NR	NR	NR	NR
SunHo Lee	2021	South Korea	Retrospective cohort	2177	2177	62.9 ± 6.5	NR	120	44	44	74	NR	592
J.Song	2022	Singapore	Retrospective cohort	159	159	66.0 ± 8.0	28.1 ± 5.3	24	NR	NR	3	NR	2
J.Erard	2023	France	Case–control	73	73	64.9 ± 9.1	29.1 ± 5.5	159.6 ± 39.6	14	6	1	NR	8
Ahmed M. Abdelaal	2023	Egypt	Case series	33	31	64.6 ± 4.5	28 ± 4.1	51.6 ± 13.2	4	0	2	NR	7
Francesco Pardo	2024	Italy	Retrospective cohort	383	383	NR	NR	116.4 ± 60.0	28	28	NR	NR	NR
Tianshun Xie	2024	Netherlands	Retrospective cohort	65	65	62.6 ± 6.0	31.4 ± 5.3	12	NR	NR	NR	NR	0

*Abbreviations: SD* standard deviation, *OA* osteoarthritis, *ACL* anterior cruciate ligament, *BMI* body mass index, *MRI* magnetic resonance imaging, *HKA* hip-knee-ankle angle, *MPTA* medial proximal tibial angle, *NR* not reported

Single-arm proportions were pooled using a random-effects generalized linear mixed model (GLMM) with a logit transformation (PLOGIT) to ensure that estimated proportions and their confidence intervals remained bounded between 0 and 1, particularly for outcomes with very low event rates. Between-study variance was estimated within the GLMM framework, and 95% confidence intervals (CIs) were calculated using the Hartung–Knapp adjustment.

For continuous variables (e.g., age), mean differences (MD) with 95% CI were calculated when preoperative and postoperative values were available. For binary outcomes including revision, reoperation, infection, and complication rates, single-arm pooled proportions with corresponding 95% CIs were estimated. The forest plots for UKA and TKA represent pooled single-arm estimates of outcomes rather than direct comparative effect sizes. The analyses of the current study are descriptive and exploratory in nature, intended to summarize outcome distributions within each treatment group rather than to infer direct treatment superiority.

Between-study heterogeneity was quantified with Cochran’s Q and the I^2^ statistic (low: < 25%; moderate: 25–50%; high: > 50%). Given anticipated clinical and methodological variation, a random-effects model (the restricted maximum likelihood estimator) was used as the default approach. A subgroup analysis was performed to assess the impact of the publication year on revision rates.

Publication bias using Egger’s test was assessed only for outcomes including at least 10 studies. Outcomes with fewer studies were considered non-assessable for publication bias and were interpreted descriptively. Funnel plots were provided to visualize potential asymmetry when at least 3 studies were available. The exploratory post-2020 temporal analysis was performed post hoc following data extraction and was not prespecified in the study protocol.

## Results

### Study selection

**Fig. 1:**
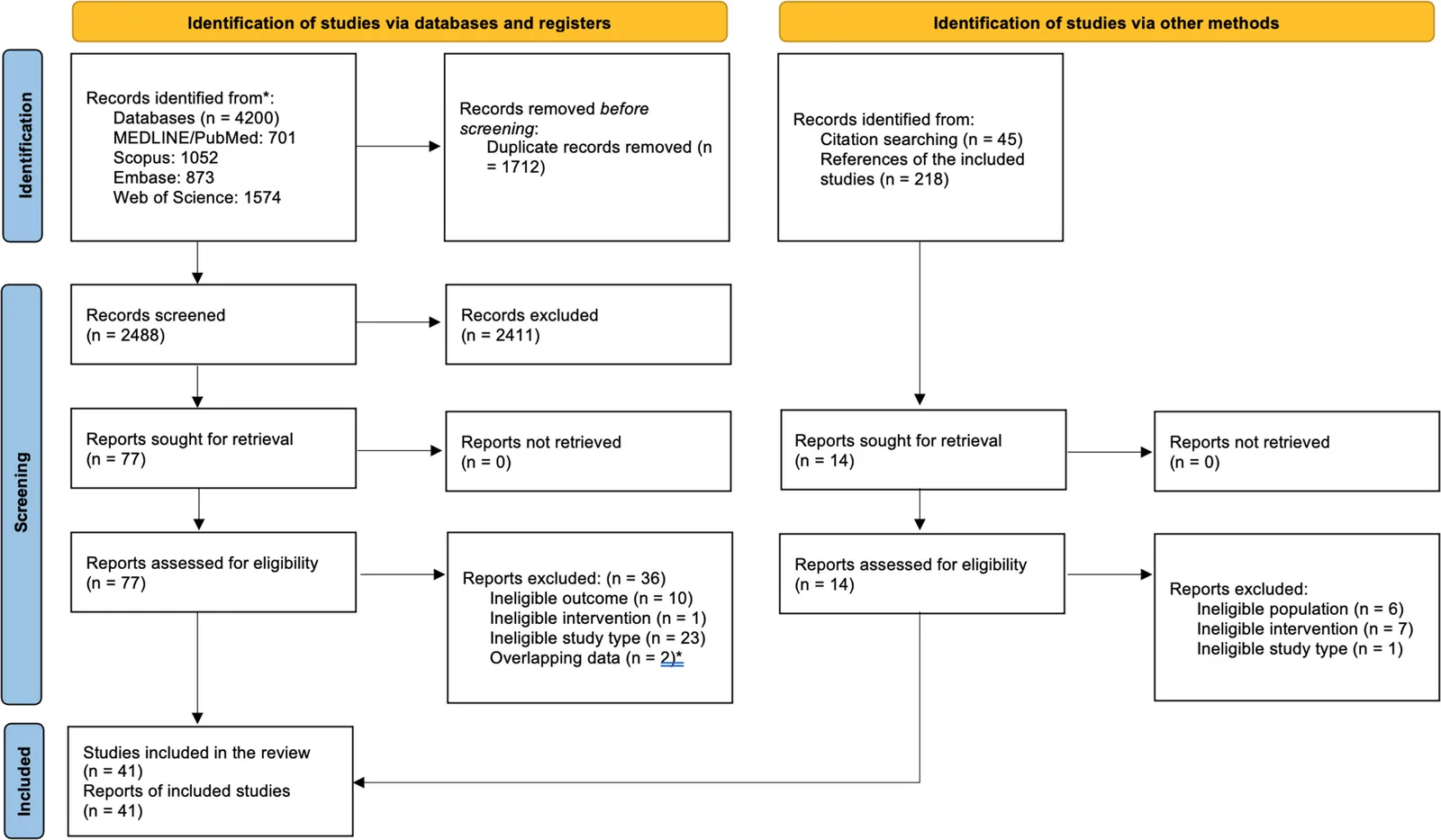
PRISMA diagram of the studies. (* signifies the study with overlapping data)

The database search initially identified 4,200 records, of which 1,712 were duplicates and subsequently removed. The remaining 2,488 records were screened based on their titles and abstracts using Rayyan. Following this preliminary screening, the full texts of 77 articles were reviewed in detail for eligibility. During this process, we identified two article pairs [23, 24] and [25, 26] published in the same year by the same center; to clarify whether these studies represented overlapping patient populations, we contacted the corresponding authors, who confirmed that the cohorts were identical. Accordingly, the two studies with lower sample sizes [23, 26] were excluded to avoid duplication. Ultimately, 41 studies met the inclusion criteria and were incorporated into the systematic review. The study selection process is illustrated in Fig. 1.

### Study characteristics

Forty-one studies (6 UKA-only, 33 TKA-only, and 2 comparative studies contributing to both groups), comprising 6,948 knees (226 knees in UKA and 6,722 knees in TKA), were separately analyzed. Among the 41 studies included in the review, 2 were comparative studies [24, 27] directly comparing outcomes of UKA and TKA following HTO.

As revision rate is a time-dependent outcome, UKA and TKA revision rates could not be directly compared because follow-up duration differed substantially. Due to the limited number of comparative studies, we categorized the included studies based on the reported outcomes for each category in separate groups. The included studies were published between 1987 and 2025. They originated in various countries, including France (5), the United States (5), Germany (4), England (4), South Korea (3), Japan (2), Singapore (2), Italy (2), Canada (2), Sweden (2), Belgium (1), Spain (1), India (1), Finland (1), Greece (1), Denmark (1), the Netherlands (1), Argentina (1), Egypt (1), and Romania (1).

### Quality appraisal

**Fig. 2:**
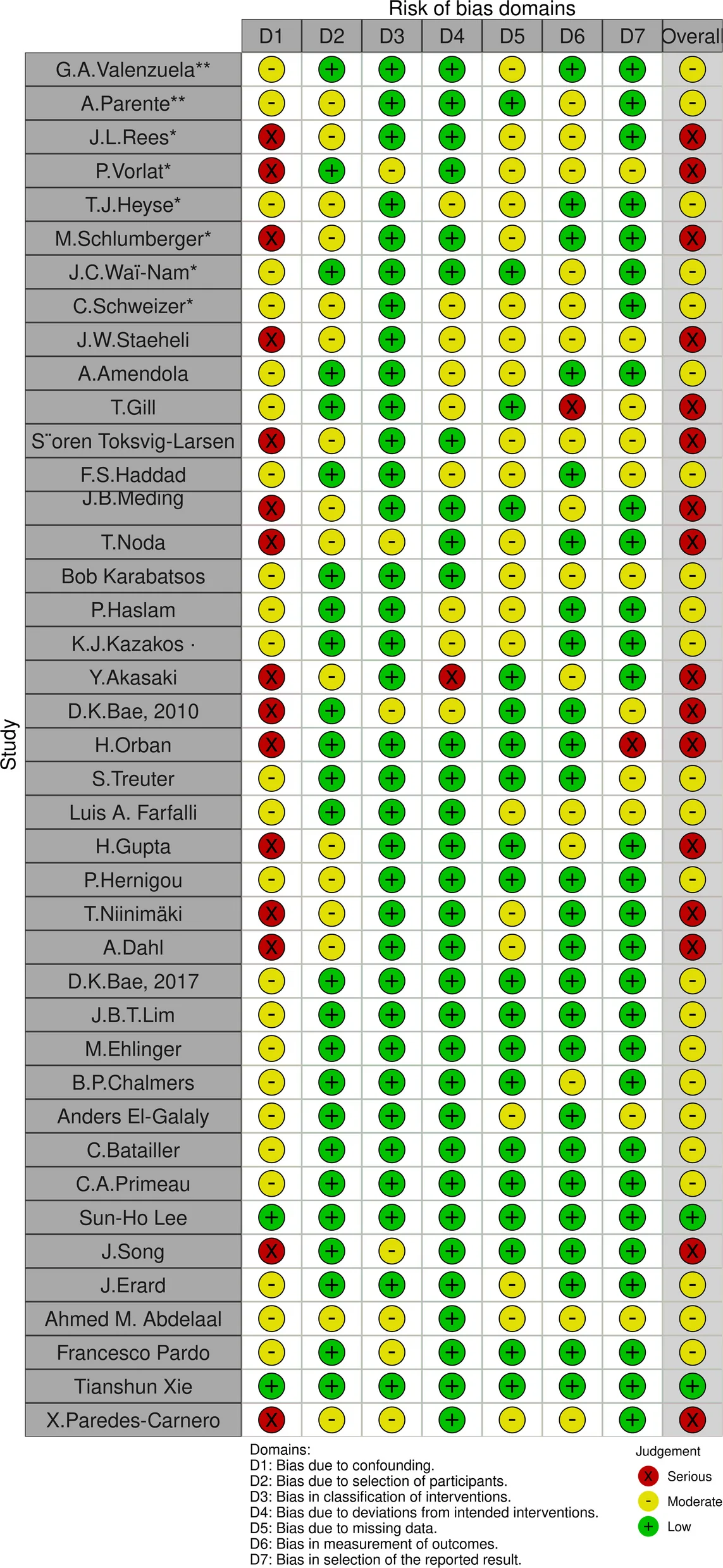
Quality appraisal of the included studies

The review of the included studies regarding their quality revealed 16 studies [10, 25, 28–41] with a serious risk of bias and 23 studies [24, 27, 42–62] with a moderate risk of bias. The remaining 2 studies [63, 64] had a low risk of bias. The serious risk of bias was mostly due to the inadequate control of confounding variables. Also, high-risk drivers (hardware status, alignment reporting) were a source of bias in the included studies. The methodological quality of the included case series, as assessed using the MINORS instrument, was moderate, with total scores ranging from 9 to 12 out of a maximum of 16. All included case series clearly stated their aims and reported appropriate endpoints and follow-up, whereas none reported prospective data collection or a prospective sample size calculation. Figure 2 represents the traffic light plot for quality appraisal of included studies. Supplementary Table S1 represents the quality appraisal of the included case series.

### HTO Conversion to UKA: patient characteristics and outcome data

In the UKA group, 8 studies [10, 24, 27–29, 42–44] were included, published between 2001 and 2025. The study designs comprised three case-control studies, two case series, and three cohort studies. Across these studies, a total of 218 patients, accounting for 226 knees, were analyzed. The mean age of the participants was 61.7 years. The mean BMI of the participants was 26.8. Among the reported data, 88 participants were male. The mean postoperative follow-up length was 64.2 months and ranged from 51.6 to 120.0 months. Table 1 represents the baseline characteristics and outcomes of the UKA group.

**Fig. 3:**
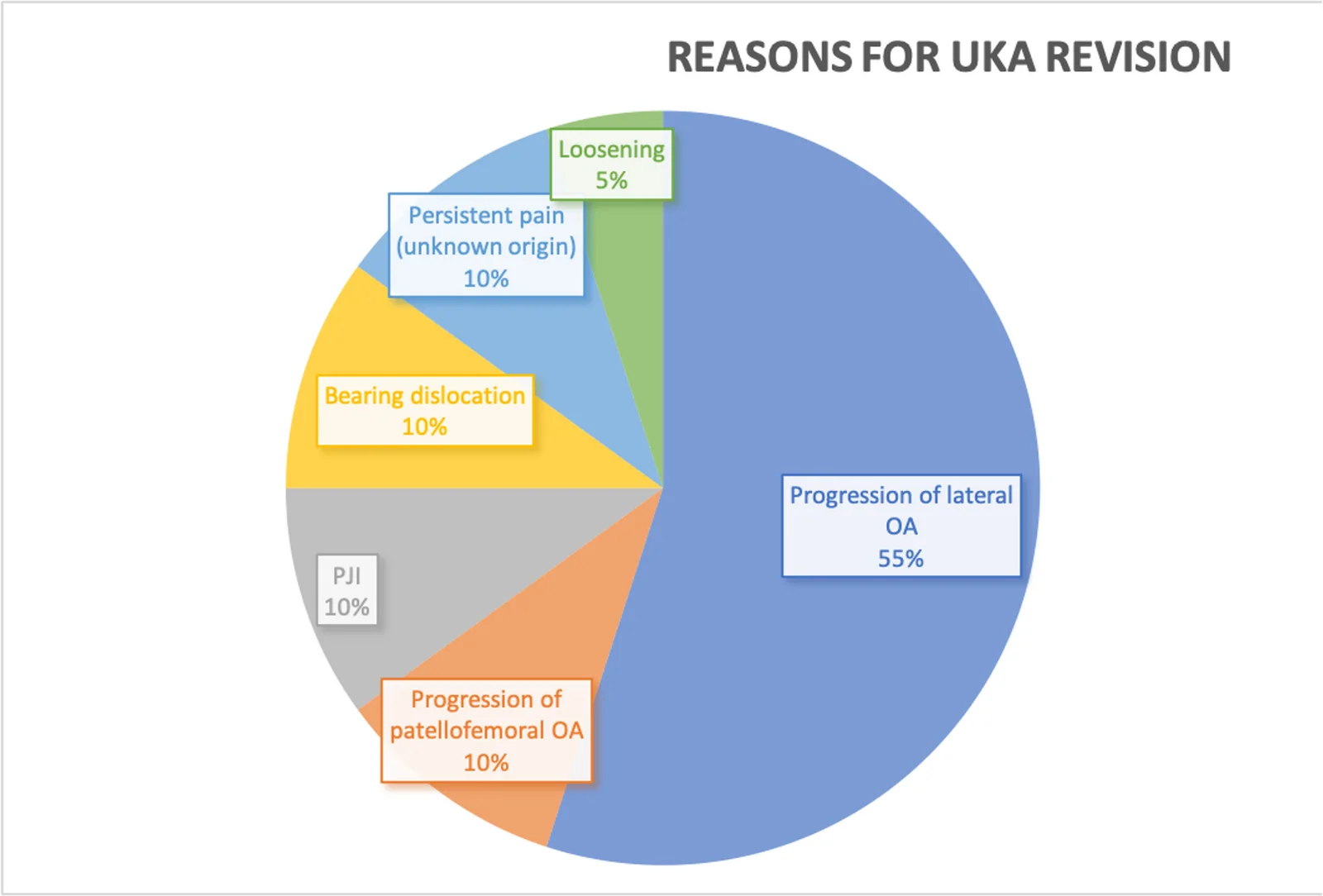
Pie chart of reasons for revision in UKA after HTO. The chart presents the distribution of reasons for revision based on a total of 22 revision events extracted from 8 included studies [10, 24, 27–29, 42–44]. The six categories shown represent all revision indications reported in the included studies, including lateral compartment progression, progression of patellofemoral OA, PJI, bearing dislocation, persistent pain, and loosening, which were derived directly from the extracted data

**Fig. 4:**
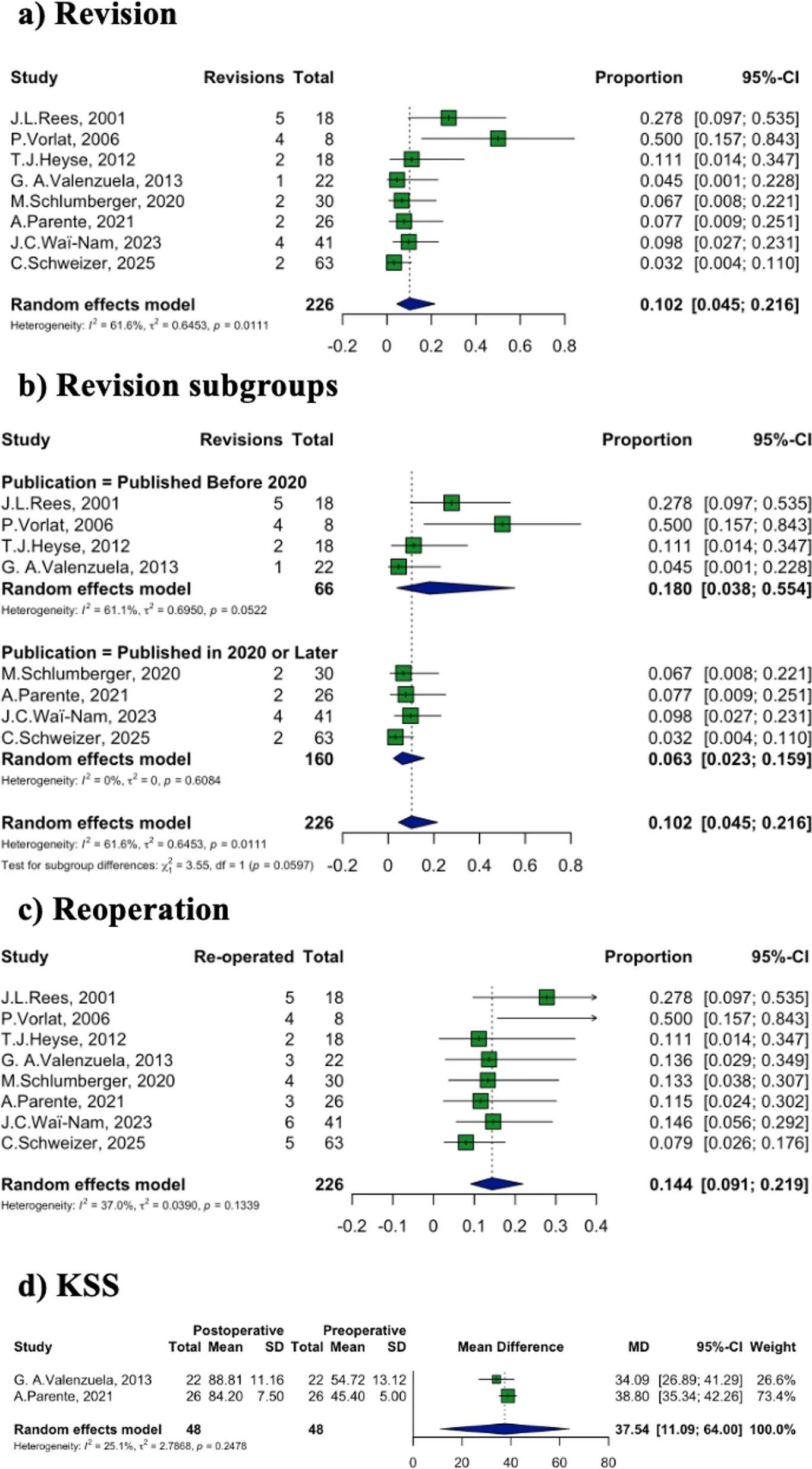
Forest plots of outcomes in UKA after HTO. (a) Revision, (b) Revision subgroups, (c) Reoperation, and (d) KSS score. *The forest plots represent single-arm pooled estimates of outcome proportions within each group, not direct comparative effect sizes, and that they should be interpreted as descriptive and exploratory

Two studies [29, 43] reported postoperative infections in this group, while 6 studies [10, 24, 27, 29, 42, 43] reported other surgical complications. The pooled rate of outcomes in this group demonstrated that, across the included studies, the overall rate of revision surgery (conversion to TKA) was 10.2% (95% CI = 4.5–21.6%, I^2^ = 61.6%) (in 226 knees), the rate of reoperations and complications was 14.4% (95% CI = 9.1–21.9%, I^2^ = 37.0%) (in 226 knees) and 15.3% (in 163 knees), respectively. Figure 3 provides the reasons for UKA revisions in the included studies. Figure 4 represents the forest plots of reoperation, revision, and KSS outcomes. Corresponding funnel plots are available in the Supplementary file.

Two studies [24, 27] reported the KSS score in the UKA group (Fig. 4). The mean difference in the KSS score ranged between 34.0 and 38.8. Two studies [27, 29] evaluated the improvement in OKS, with MD ranging from 17.3 to 21.4. One study [24] reported the UCLA and WOMAC scores as 2.9 and 32.4, respectively, following UKA. Regarding radiographic outcomes for UKA, one study [27] reported a postoperative Insall–Salvati ratio of 0.86 ± 0.17.

### HTO Conversion to TKA: patient characteristics and outcome data

A total of 35 studies, published between 1987 and 2024, provided data on HTO conversion to TKA. These studies covered 6,632 patients and 6,722 knees. Among the provided data, 2396 were male. The mean age of the participants was 63.6 years, and the mean BMI was 30.7. The average follow-up in the TKA group was 102.4 months (range: 12 to 180 months) (Table 2).

**Fig. 5:**
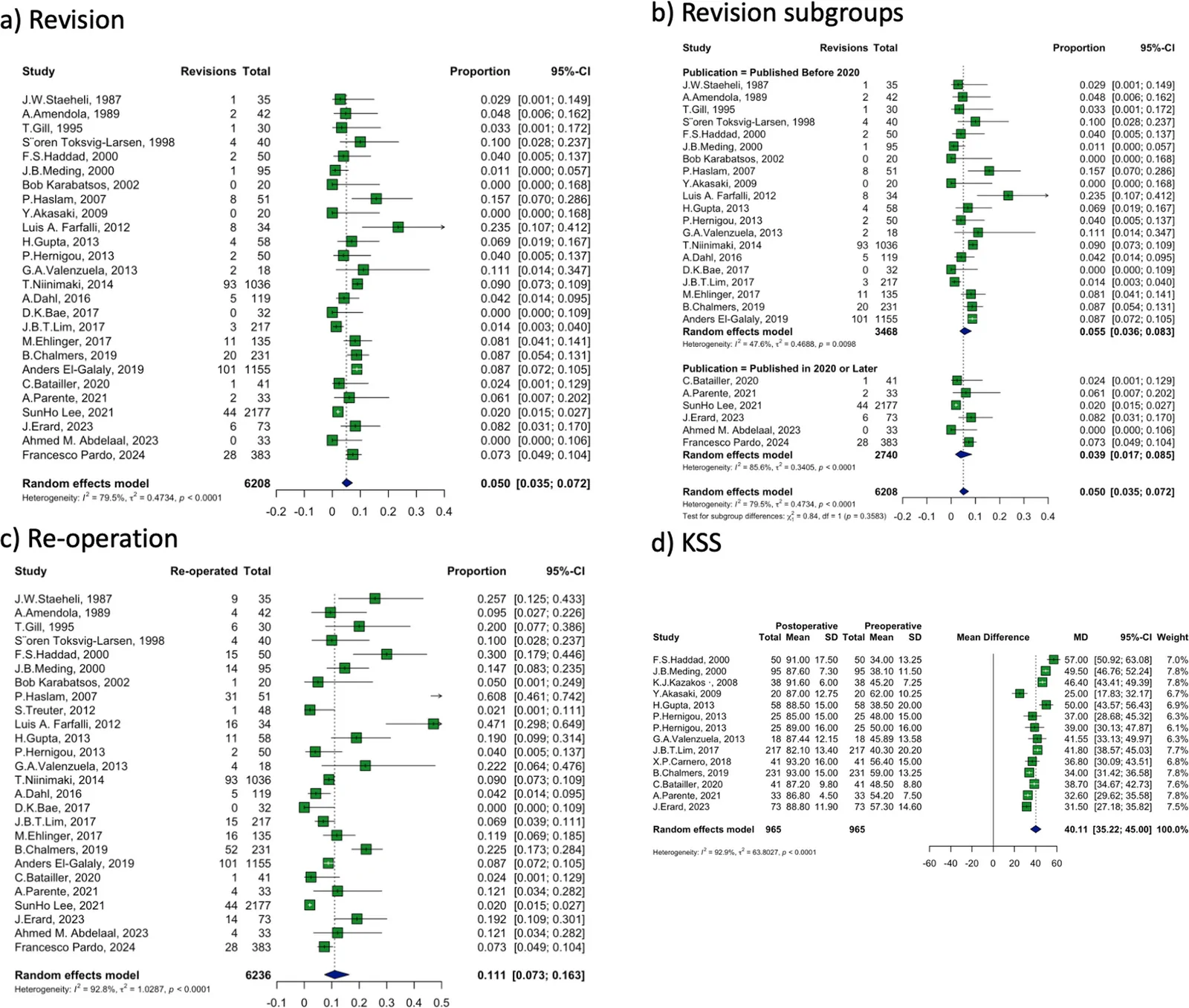
Forest plots of outcomes in TKA after HTO. (a) Revision, (b) Revision subgroups, (c) Re-operation, (d) KSS. *The forest plots represent single-arm pooled estimates of outcome proportions within each group, not direct comparative effect sizes, and that they should be interpreted as descriptive and exploratory. **The pooled point estimates in Fig. 5c and Fig. 5 d should be interpreted only as summaries of a wide underlying distribution and not as precise single values. *** The study by Hernigou et al. (2013) in Fig. 5 d was designed as a two-arm study, reporting two distinct cohorts that were included separately in the meta-analysis

The meta-analysis showed a pooled infection rate of 2.9% (in 2107 knees) following TKA. Pooled reoperation and revision rates in the TKA group were 11.1% (95% CI = 7.3–16.3%, I^2^ = 92.8%) (in 6236 knees) and 5.0% (95% CI = 3.5–7.2%, I^2^ = 79.5%) (in 6208 knees), respectively (Table 2). Forest plots for the reoperation and revision outcomes in the TKA group are presented in Fig. 5. The pooled point estimates of high heterogeneity (reoperation rate and KSS score) should be interpreted only as summaries of a wide underlying distribution and not as precise single values. Corresponding funnel plots are available in the Supplementary file.

The meta-analysis of the findings regarding the HSS score revealed a statistically significant improvement following TKA. The pooled estimate of HSS score was 40.1 (95% CI 29.2–51.0). Similarly, the results from the KSS questionnaire improved following TKA by a pooled estimate of 40.8 points (95% CI 36.0–45.6). The OKS demonstrated a substantial pooled improvement of 16.9 points (95% CI 16.02–20.05) following TKA surgery. The postoperative Insall–Salvati ratio, reported in seven studies, showed a pooled estimate of 1.11 (95% CI 0.9–1.2).

### Direct comparative studies between UKA and TKA after HTO

Only two papers [24, 27] performed a direct comparison between HTO conversion to UKA vs. TKA, both of which reported comparable results regarding functional scores, revision rates, and surgical complications (Table 3). Given the small number of comparative studies, a separate quantitative synthesis (e.g., pooled RR/OR or dedicated forest plot) was not methodologically appropriate due to insufficient statistical power and risk of misleading interpretation. Therefore, the findings from these studies are presented descriptively and reported separately.

**Table 3:** Summary table of comparative studies

Author, year	Sample size	Follow-up	Revision	Reoperation	Infection	Changes in KSS score	Complications	Authors' reported conclusions
Valenzuela, 2013	UKA after HTO: 22 TKA after HTO: 18	UKA after HTO: 77.6 months TKA after HTO: 99 months	UKA after HTO: 1 TKA after HTO: 2	UKA after HTO: 3 TKA after HTO: 4	UKA after HTO: not reported TKA after HTO: not reported	UKA after HTO: 34.09 TKA after HTO: 41.55	UKA after HTO: 3 TKA after HTO: 4	The results for the patients who underwent UKA after HTO demonstrated good functional outcomes after six years of follow-up and provided results comparable to those of TKA after HTO or primary UKA
Parente, 2021	UKA after HTO: 26 TKA after HTO: 33	UKA after HTO: 93.6 months TKA after HTO: 134.4 months	UKA after HTO: 2 TKA after HTO: 2	UKA after HTO: 2 TKA after HTO: 2	UKA after HTO: 0 TKA after HTO: 0	UKA after HTO: 38.8 TKA after HTO: 32.6	UKA after HTO: 2 TKA after HTO: 2	Concerning clinical relevance, selected patients with failed HTO may benefit from UKA instead of TKA, thus allowing the advantages of a minimally invasive procedure without affecting clinical and functional recovery but rather delaying more invasive surgeries

*Abbreviations: KSS* knee society score, *TKA* total knee arthroplasty, *UKA* unicompartmental knee arthroplasty, *NR* not reported

### Separate analysis of the contemporary literature

**Fig. 6:**
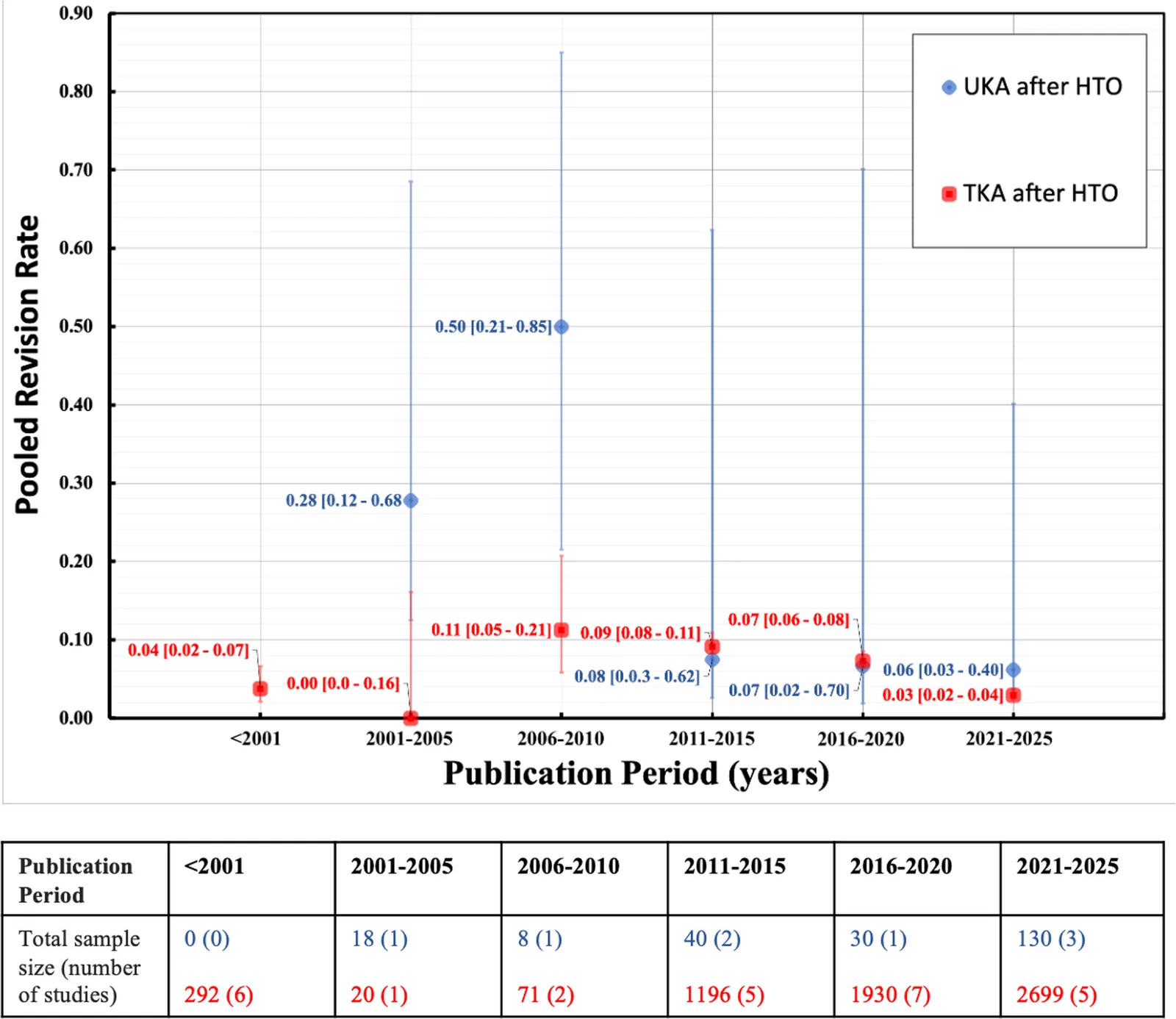
Temporal trend of revision rates in UKA and TKA after HTO. This figure is based on publication year rather than surgical year and represents an exploratory post hoc analysis

Careful assessment of the reported revision rates of UKA after HTO demonstrated a temporal trend (Fig. 6). Recent studies have reported lower revision rates compared to older studies (3% in 2025 [44] compared to 28% in 2001 [10]). As a result, a separate analysis of the recent studies published from January 2020 was employed as a pragmatic approach to explore a contemporary vs. historical temporal trend in outcomes and to obtain a clearer understanding of recent HTO conversion outcomes. The results showed a revision rate of 6.3% and 3.9% for post-HTO UKA and TKA, respectively (Figs. 4 and 5). However, the between-period difference was not statistically significant (UKA between-group *p*-value = 0.0597, TKA between-group *p*-value = 0.3583).

## Discussion

This systematic review and meta-analysis sought to evaluate the outcomes of UKA and TKA following HTO. Historically, a previous HTO was regarded as a contraindication to UKA due to concerns regarding altered alignment, ligament imbalance, and compromised bone stock. However, recent advances in implant design, imaging, and surgical techniques have led to a reappraisal of this assumption. Our findings indicate that UKA, when performed in appropriately selected patients, yields acceptable functional and revision outcomes, prompting reconsideration of historical contraindications in selected cases.

Across the included studies, UKA demonstrated substantial improvement in functional outcomes, with KSS MD ranging between 34 and 39 points, and OKS MD between 17.3 and 21.4. These findings indicate that, in carefully selected patients, UKA can provide clinically meaningful functional improvements following HTO. However, interpretation of these findings should remain cautious because the available evidence is derived predominantly from retrospective observational studies with heterogeneous patient populations, surgical techniques, and follow-up durations.

Our pooled analysis demonstrated a revision rate of 10.2% (95% CI 4.5–21.6%) and a reoperation rate of 14.4% (95% CI 9.1–21.9%) following UKA after HTO, with PJI rates ranging from 0 to 3.3%. In comparison, the TKA group demonstrated revision, reoperation, and PJI rates of 5.0 (95% CI 3.5–7.2%), 11.1 (95% CI 7.3–16.3%), and 2.9%, respectively. In separate pooled analyses, UKA demonstrated revision, reoperation, and infection rates that numerically differed from those reported for TKA; however, these findings should be interpreted cautiously because they were derived from non-comparative and single-arm syntheses. Importantly, no study reported mortality, and the overall complication rates were low in both groups.

Early UKA series in patients with failed HTO [10, 28] documented revision rates as high as 28 and 50%, and consequently advised against performing UKA in patients with prior HTO. In contrast, contemporary studies [29, 44] report outcomes approaching those of primary UKA (Fig. 4). This improvement likely reflects advances in implant design, enhanced understanding of UKA biomechanics, greater surgical experience, and more rigorous patient selection. Refined alignment and ligament-balancing strategies, and better management of residual deformity or retained hardware after HTO may also have contributed to the lower revision rates reported in recent cohorts. Furthermore, modern technologies such as computer-assisted navigation and robotic-assisted implantation may improve component positioning and soft-tissue balancing in technically challenging post-HTO knees. Collectively, these findings suggest that in the modern surgical era, UKA represents a technically feasible option for arthroplasty following HTO in appropriately selected patients for whom unicompartmental replacement may be sufficient. However, the available evidence remains insufficient to determine the comparative effectiveness of UKA relative to TKA, and treatment decisions should continue to be individualized based on patient characteristics, residual deformity, and surgeon experience.

It is important to emphasize that these findings should not be interpreted as a recommendation to perform UKA in every patient with a failed HTO. Scrutiny of the reported indications in the literature suggests that similar indications to primary UKA may be used. Patients with medial bone-on-bone osteoarthritis and a history of HTO can be considered for UKA if there are no signs of bone-on-bone lateral osteoarthritis, ACL or MCL insufficiency, and patellofemoral bone loss, grooving, or subluxation. Conversely, patients with overcorrection into valgus, advanced tricompartmental degeneration, or ACL/MCL insufficiency are poor candidates for UKA and remain better suited for TKA [12]. After HTO, the tibia is often in relative valgus, the MPTA is increased, patellar height altered, and ligament stability reduced. These scenarios predispose to UKA failure via lateral progression or tibial component malposition, but in appropriately selected patients with acceptable MCL integrity, absence of severe lateral OA, and no sign of deformity overcorrection, UKA may be considered a treatment option.

Another important consideration when interpreting the favorable contemporary outcomes of UKA after HTO is the high degree of surgical experience and case concentration reflected in the included studies. The majority of UKA series originated from specialized, high-volume centers with established expertise in both knee osteotomy and unicompartmental arthroplasty, often led by surgeons with extensive experience in patient selection, alignment assessment, and ligament balancing. This concentration of expertise likely mitigates the technical challenges inherent to post-HTO anatomy, including altered tibial slope, residual coronal malalignment, and prior hardware [23, 24, 44]. Several studies explicitly reported stringent adherence to classic Oxford UKA indications and emphasized meticulous intraoperative correction of residual deformity, avoidance of valgus overcorrection, and conservative medial compartment filling [44]. These factors are not uniformly reproducible across low-volume or non-specialist settings and may partially explain the historically inferior results reported in earlier, less experienced series. Therefore, the improving survivorship and declining revision rates observed in recent UKA studies should be interpreted in the context of surgeon experience and institutional volume, reinforcing that UKA after HTO is a technically demanding procedure best performed by surgeons with substantial UKA expertise rather than a broadly generalizable alternative to TKA. The available evidence may not be fully generalizable to lower-volume centers or non-specialist settings, which is an important limitation for clinical translation.

Patients undergoing conversion from HTO to arthroplasty are typically younger (40–60 years) and more active than the general knee arthroplasty population, and therefore have higher functional expectations [3, 65]. Based on the current review, progression of lateral compartment OA after medial UKA remains the principal mode of failure (55%) and a major concern for surgeons. Early studies supported this concern, reporting high revision rates attributed to lateral progression of OA secondary to overcorrected HTOs. In the series by Rees et al., for example, five of eighteen knees required revision to TKA, three of which failed because of residual valgus alignment [10]. Collectively, these findings suggest that careful attention to residual alignment, particularly avoidance of valgus overcorrection, may reduce the risk of lateral compartment degeneration following UKA after HTO. Therefore, surgeons converting an HTO to UKA should avoid overstuffing the medial compartment, using excessively thick inserts, or creating valgus overcorrection, as these factors increase the risk of lateral progression of OA.

An additional biomechanical consideration following HTO is alteration of the posterior tibial slope (PTS). Changes in PTS are well recognized after osteotomy, particularly following opening-wedge HTO, where unintended increases in slope may occur if anterior and posterior gap management is not carefully controlled [66]. Altered PTS can influence tibiofemoral kinematics, ligament tension, and contact mechanics, potentially creating additional technical challenges during subsequent UKA [67]. These considerations may be particularly relevant for mobile-bearing UKA designs, as excessive slope and abnormal sagittal plane mechanics have been associated with an increased risk of bearing instability or dislocation [68]. Consequently, careful preoperative assessment of residual tibial morphology, including coronal alignment and sagittal slope, may be important when evaluating patients for UKA after HTO. In cases with substantial alteration of tibial anatomy, fixed-bearing implants may offer theoretical advantages by reducing the risk of bearing-related complications, although the available evidence remains insufficient to establish definitive implant-selection recommendations. Future studies should report postoperative alignment parameters, including PTS, and evaluate their relationship with implant survivorship and failure mechanisms following UKA after HTO.

A decision must be made regarding whether to retain, partially remove, or completely extract prior hardware. Regardless of the approach, the surgeon should remain vigilant for indolent infection and obtain appropriate clinical assessments and laboratory investigations before proceeding. Furthermore, intraoperative management of residual hardware, previous osteotomy sites, and altered tibial slope and/or patellar height must be approached cautiously to mitigate the risks of bone fracture and component malposition. Notably, several authors [42, 44] recommend the use of fixed-bearing implants, which better tolerate minor residual medial laxity following HTO compared with mobile-bearing designs.

Our review builds upon and extends the findings of Legnani et al., who previously analyzed UKA outcomes after failed HTO but did not directly compare them with TKA outcomes [13]. In this updated meta-analysis, we incorporate a broader and more contemporary evidence base, including studies published up to 2025, and report outcomes for both UKA and TKA following HTO. This allows for an indirect, descriptive comparison of functional outcomes, complication rates, and revision rates across procedures. By synthesizing these newer data, our study provides an updated perspective on the feasibility and potential role of UKA in carefully selected patients, highlighting trends in revision rates and functional recovery in the modern surgical era.

Despite the lack of clarity to provide a detailed comparison between baseline patient characteristics of UKA versus TKA groups, reported indications and patient selection criteria imply the presence of major differences and heterogeneity. While UKA studies usually consider MCL/ACL integrity and isolated unicompartmental severe osteoarthritis as surgical indications, these criteria have not been used for patient selection in TKA studies. Furthermore, only two comparative studies exist in the literature. As a result, we were not able to perform a direct comparison between UKA and TKA outcomes in patients with failed HTO and merely provided a comprehensive view of existing literature. This study is an attempt to urge knee surgeons around the world to perform prospective comparisons between these two groups in order to shed more light on this dilemma.

The limitations of this review must be acknowledged. The limited number of direct comparative studies precluded a formal head-to-head meta-analysis between UKA and TKA. In addition, follow-up durations varied widely, with only a few studies exceeding 10 years, thereby limiting long-term survivorship conclusions. Also, the lower revision rates observed in post-2020 publications may be partially attributable to shorter follow-up duration in more recent studies and should not be interpreted solely as reflecting improvements in surgical technique, implant design, or patient selection. Most revision HTOs converted to UKA occur in niche expert centers, whereas TKA remains the pragmatic workhorse in routine practice, which may result in selection bias. Consequently, these findings may not be directly generalizable to lower-volume centers or non-specialist settings. Single-surgeon comparative studies are required to remove the potential effect of surgeon experience. The lack of comparison in the reported parameters hindered our ability to perform subgroup analyses regarding ACL integrity and postoperative alignment to determine a specific cutoff at which conversion to UKA becomes inappropriate. The marked size difference between UKA and TKA cohorts limits the stability of comparisons, as rare events weigh more heavily in the smaller UKA group. Although our search strategy included the abbreviation “UKA”, it did not explicitly include the full-text terms “unicompartmental” or “unicondylar” knee arthroplasty. Consequently, studies indexed exclusively using these terms without the abbreviation may have been missed, although manual reference screening was also performed to minimize this risk. Finally, heterogeneity in surgical technique (lateral closing wedge HTO vs. medial opening wedge HTO), implant design, alignment correction after HTO, and ligament balance reflects real-world practice but may have influenced reported outcomes.

Future research should prioritize well-designed prospective comparative cohorts/registry-based matched analyses with standardized outcomes directly evaluating UKA versus TKA in patients with prior HTO to address the limitations of the current evidence base, which is predominantly retrospective and derived from single-arm studies. From a clinical perspective, the current evidence suggests that UKA may represent a reasonable option in carefully selected patients treated in experienced centers, but should not be generalized as equivalent to TKA.

## Conclusions

This study suggests that UKA after HTO should not be considered an absolute contraindication and may have a role in a carefully selected subset of patients with isolated medial osteoarthritis, sufficient MCL, and acceptable residual alignment. However, these findings should be interpreted cautiously, as the current evidence base consists predominantly of retrospective observational studies. Accordingly, the present findings should be viewed as hypothesis-generating rather than as evidence establishing equivalence or comparative effectiveness between UKA and TKA after failed HTO. Future prospective comparative studies are needed to better define the effectiveness of UKA relative to TKA, refine patient selection algorithms, and clarify the influence of residual deformity on outcomes. These studies should incorporate standardized definitions of revision, reoperation, and complications; consistent reporting of HTO technique and alignment parameters; and sufficient follow-up to evaluate mid- and long-term implant survivorship.

## Supplementary Information

Supplementary Material 1.

## Data Availability

The datasets generated and analyzed during the current study are available from the corresponding author upon reasonable request.
