# Supplementary material for: Unicompartmental knee arthroplasty after failed high tibial osteotomy in appropriately selected patients: a systematic review and meta-analysis
Source: Arthroplasty. 2026 Oct 5;8:74. doi: 10.1186/s42836-026-00434-2 (PMC13637293; doi:10.1186/s42836-026-00434-2)
Supplement: Supplementary file 1 [file 42836_2026_434_MOESM1_ESM.docx]

**Supplementary material**

**Search Strategy**

Scopus 1052

TITLE-ABS-KEY ((tka OR “Knee Arthroplasty” OR “Knee Replacement” OR uka) AND (hto OR “High tibia* osteotom*” OR “proximal tibia* osteotom*” OR “tibia* proximal corrective osteotom*” OR “tibia* proximal osteotom*”))

Embase 873

(tka:ti, ab, kw OR “knee arthroplasty”:ti, ab, kw OR “knee replacement”:ti, ab, kw OR uka:ti, ab, kw) AND (hto:ti, ab, kw OR “high tibia* osteotom*”:ti, ab, kw OR “proximal tibia* osteotom*”:ti, ab, kw OR “tibia* proximal corrective osteotom*”:ti, ab, kw OR “tibia* proximal osteotom*”:ti, ab, kw)

Pubmed 701

(tka[Title/Abstract] OR “Knee Arthroplasty”[Title/Abstract] OR “Knee Replacement”[Title/Abstract] OR uka [Title/Abstract]) AND (hto[Title/Abstract] OR “High tibia* osteotom*”[Title/Abstract] OR “proximal tibia* osteotom*”[Title/Abstract] OR “tibia* proximal corrective osteotom*”[Title/Abstract] OR “tibia* proximal osteotom*” [Title/Abstract])

Web of Science 1574

(tka OR “Knee Arthroplasty” OR “Knee Replacement” OR uka) AND (hto OR “High tibia* osteotom*” OR “proximal tibia* osteotom*” OR “tibia* proximal corrective osteotom*” OR “tibia* proximal osteotom*”) (Topic)

Funnel plot for re-operation in UKA group

Funnel plot for revision in UKA group

Funnel plot for re-operation in TKA group

Funnel plot for revision in TKA group

Funnel plot for KSS in TKA group

Forest plot for infection in TKA group

Funnel plot for infection in TKA group

Forest plot for HHS in TKA group

Funel plot for HHS in TKA group

Forest plot for OKS in TKA group

Forest plot for OKS in TKA group

Forest plot for Insall-Salvati in TKA group

Funnel plot for Insall-salvati in TKA group

Supplementary Table S1. Methodological quality assessment of the included case series studies according to the Methodological Index for Non-Randomized Studies (MINORS)

| **Study** | **Aim** | **Consecutive patients** | **Prospective data collection** | **Appropriate endpoints** | **Unbiased assessment** | **Follow-up** | **Loss to follow-up** | **Sample size calculation** | **Total (/16)** |
| --- | --- | --- | --- | --- | --- | --- | --- | --- | --- |
| **M. Schlumberger, 2020** | 2 | 2 | 0 | 2 | 2 | 2 | 1 | 0 | 11 |
| **J. C. Wai Nam, 2023** | 2 | 2 | 0 | 2 | 2 | 2 | 1 | 0 | 11 |
| **Y. Akasaki, 2009** | 2 | 2 | 0 | 2 | 2 | 2 | 1 | 0 | 11 |
| **H. Orban, 2011** | 2 | 1 | 0 | 2 | 1 | 2 | 1 | 0 | 9 |
| **S. Treuter, 2012** | 2 | 2 | 0 | 2 | 2 | 2 | 2 | 0 | 12 |
| **A. M. Abdelaal, 2023** | 2 | 2 | 0 | 2 | 2 | 2 | 2 | 0 | 12 |
